# Diagnostic Utility of Serum Sodium Compared With C-reactive Protein and Total Leukocyte Count for Predicting Appendiceal Perforation and Clinical Outcomes in Adult Patients With Acute Appendicitis: A Prospective Observational Study

**DOI:** 10.7759/cureus.110359

**Published:** 2026-06-06

**Authors:** Deepak Kumar, Harsh Gosain, Samir Anand, Priyanjana Krishna

**Affiliations:** 1 Department of General Surgery, Maharishi Markandeshwar Medical College and Hospital, Solan, IND; 2 Department of Anatomy, Maharishi Markandeshwar Medical College and Hospital, Solan, IND

**Keywords:** acute appendicitis, appendiceal perforation, biomarkers, c-reactive protein, diagnostic utility, hyponatremia, predictive biomarker, serum sodium, total leukocyte count

## Abstract

Background

Early identification of complicated appendicitis remains a clinical challenge despite advances in diagnostic methods. Appendiceal perforation is associated with increased morbidity, prolonged hospitalization, and adverse postoperative outcomes. Conventional inflammatory markers such as C-reactive protein (CRP) and total leukocyte count (TLC) have shown variable utility in predicting disease severity. Recently, serum sodium has emerged as a potential biomarker for identifying complicated appendicitis. The present study evaluated the utility of serum sodium as a predictor of appendiceal perforation and compared its performance with CRP and TLC.

Methods

This prospective observational study was conducted in the Department of General Surgery at Maharishi Markandeshwar Medical College and Hospital, Solan, Himachal Pradesh, India. A total of 72 adult patients with acute appendicitis diagnosed using combined clinical, laboratory, imaging, intraoperative, and histopathological findings were enrolled. Serum sodium, CRP, TLC, and other laboratory parameters were measured at admission before surgical intervention. Cases were categorized as non-perforated and perforated appendicitis. Diagnostic performance was evaluated using receiver operating characteristic (ROC) analysis, and correlations with hospital stay and postoperative complications were assessed.

Results

The mean age of participants was 41.1±15.26 years, and males constituted 68.1% of the cohort. Perforated appendicitis was identified in 15.3% of patients and was associated with higher age (49.55±18.30 vs. 39.59±14.31 years) and lower serum sodium levels (133.63±3.80 vs. 137.92±2.88 mEq/L) compared with non-perforated appendicitis. Hyponatremia showed a strong association with perforation. ROC analysis demonstrated good discriminatory ability of serum sodium (area under the curve or AUC=0.803), with a sensitivity of 63.6% and a specificity of 91.8% at a cutoff value of 135 mEq/L, indicating high specificity but modest sensitivity for predicting perforated appendicitis. CRP showed the best overall diagnostic accuracy (AUC=0.807), whereas TLC demonstrated limited predictive utility (AUC=0.626). Patients with perforated appendicitis had longer hospital stay (5.0±2.14 vs. 3.44±1.09 days) and higher postoperative complication rates (45.5% vs. 3.3%) compared to those with non-perforated appendicitis. Serum sodium showed a negative correlation with hospital stay (r=−0.261) and also demonstrated predictive ability for postoperative complications (AUC=0.733).

Conclusions

Serum sodium is a readily available and cost-effective adjunctive biomarker associated with appendiceal perforation and post-operative complications in patients with acute appendicitis. Although CRP demonstrated superior overall diagnostic accuracy, serum sodium may serve as a useful adjunctive biomarker in risk stratification of acute appendicitis. Incorporation of serum sodium into multimodal risk assessment strategies may facilitate earlier recognition of complicated disease; however, larger multicenter studies are required before routine clinical implementation can be recommended.

## Introduction

Acute appendicitis is among the most common surgical emergencies worldwide, affecting approximately 1% of the global population [1]. It is characterized by inflammation and obstruction of the vermiform appendix and commonly presents with abdominal pain, nausea, vomiting, and fever [2]. Other complications of acute appendicitis include localized abscess formation, wound infection, sepsis, bowel obstruction, prolonged hospitalization, and increased postoperative morbidity [3]. The lifetime risk of acute appendicitis ranges from 7% to 9%, with a higher incidence among young individuals, particularly male subjects. Diagnosis is traditionally based on clinical history and physical examination [4]. However, despite advances in clinical assessment, even experienced surgeons achieve a diagnostic specificity of approximately 85% [5]. Furthermore, conventional diagnostic approaches may be less reliable in identifying complicated appendicitis, which is associated with increased morbidity and mortality [6].

Serum sodium levels have recently gained attention as a potential biomarker in acute appendicitis. Serum sodium is routinely measured and reflects the balance between water intake and excretion in the body [7]. Its levels may be influenced by several factors, including electrolyte disturbances, renal dysfunction, medications, dehydration, and systemic inflammatory processes. In patients with acute appendicitis, serum sodium levels have been associated with multiple clinical outcomes, including hospital length of stay, complication rates, recovery time after surgery, wound infection rates, and mortality [8].

Several studies have reported that patients with complicated appendicitis exhibit lower serum sodium levels than those with uncomplicated appendicitis and healthy individuals. These findings suggest that serum sodium may reflect the severity of inflammation or degree of tissue injury in acute appendicitis. Evidence has also suggested that serum sodium levels may serve as a useful preoperative predictor of disease severity. One study reported that preoperative serum sodium levels <136 mEq/L were associated with a significantly increased risk of complicated appendicitis compared with levels >136 mEq/L (OR: 5.35; 95% CI: 3.39-8.45) [6]. Similarly, another study demonstrated that preoperative serum sodium levels <135 mEq/L were associated with an increased risk of prolonged recovery after surgery compared with levels >135 mEq/L (OR: 2.18; 95% CI: 1.32-3.57) [9].

Given these findings, the role of serum sodium as a predictive biomarker for determining the severity of acute appendicitis remains uncertain. Therefore, the present study was designed to evaluate the utility of serum sodium levels as a predictor of disease severity in patients with acute appendicitis. Although several studies have investigated hyponatremia in acute appendicitis, prospective studies with histopathological confirmation remain limited. Furthermore, evidence regarding the utility of serum sodium in predicting postoperative outcomes is scarce. Therefore, this study was undertaken to evaluate the diagnostic utility of serum sodium in predicting appendiceal perforation and postoperative outcomes and to compare its performance with C-reactive protein (CRP) and total leukocyte count (TLC).

## Materials and methods

Study design, setting, and population

This prospective observational study was conducted in the Department of General Surgery at Maharishi Markandeshwar Medical College and Hospital, Solan, Himachal Pradesh, India, over a period of one year from April 2024 to March 2025. Ethical approval was obtained from the Institutional Ethics Committee of Maharishi Markandeshwar Medical College and Hospital, Kumarhatti, Solan (MMMCH/IEC/24/848). The study included adult patients aged 18 years and above presenting with symptoms suggestive of acute appendicitis.

Sample size calculation

The sample size was calculated based on the sensitivity of serum sodium levels <135 mmol/L for predicting complicated appendicitis. A sensitivity of 94% reported by Elgendy et al. [10] was used as the reference value, while an anticipated sensitivity of 85% was assumed for the present study. The calculation was performed using a two-sided alpha error of 0.05 and a study power of 80% (β=0.20). The sample size was estimated using the formula: N = (p₀q₀ × (Zα/2 + Zβ√(p₁q₁/p₀q₀))²) / (p₁ − p₀)²; where p₀=0.94, q₀=1−p₀, p₁=0.85, q₁=1−p₁, Zα/2=1.96, and Zβ=0.84. Based on these assumptions, the minimum required sample size was calculated to be 72 participants. Therefore, 72 patients were enrolled in the study.

Participant selection criteria

The final diagnosis of acute appendicitis was established using a combination of clinical history, physical examination findings, laboratory investigations, abdominal ultrasonography, intraoperative findings, and histopathological examination. These investigations were part of the routine institutional diagnostic protocol; however, only variables relevant to the study objectives were presented to avoid unnecessary data inclusion. Although ultrasonography was performed in all patients, a few cases showed inconclusive or alternative findings but were later confirmed as acute appendicitis intraoperatively and on histopathology. Patients with chronic kidney disease or conditions known to affect serum sodium levels, such as adrenal insufficiency or liver cirrhosis, were excluded. Pregnant women, individuals unwilling or unable to provide consent, and patients with other concurrent abdominal emergencies, including acute pancreatitis or perforated peptic ulcer, were also excluded. Patients with alternative or inconclusive ultrasonography findings were not excluded if acute appendicitis was subsequently confirmed intraoperatively and on histopathological examination.

Laboratory investigations and ultrasonography

Diagnostic investigations included complete blood count (CBC), serum electrolytes, CRP, renal and liver function tests, abdominal ultrasonography, operative findings, and histopathological examination of the resected appendix. Blood samples were collected at admission and before surgical intervention using standard aseptic venepuncture techniques. Serum sodium levels were measured in the central biochemistry laboratory using the ion-selective electrode (ISE) method. The normal reference range for serum sodium was considered as 135-145 mmol/L. Additional laboratory investigations included total leukocyte count, CRP, renal function tests, liver function tests, serum electrolytes, and random blood sugar levels. CRP and other biochemical investigations were performed using automated analyzers according to standard laboratory protocols and manufacturer recommendations. Ultrasonography of the abdomen was performed in all patients according to institutional protocol to evaluate appendiceal pathology and associated inflammatory changes. All ultrasonography examinations were performed by the same consultant radiologist to minimize interobserver variability.

Classification of appendicitis severity

Cases were classified based on intraoperative and histopathological findings. Patients with appendiceal perforation, with or without gangrenous changes, were categorized as perforated appendicitis, while all remaining patients were categorized as non-perforated appendicitis.

Outcome assessment and follow-up

Outcome variables included duration of hospital stay and postoperative complications. Postoperative complications were recorded as a composite outcome variable and included surgical site infection, wound dehiscence, postoperative ileus, intra-abdominal collection, and other complications requiring medical or surgical intervention during hospitalization. Patients underwent daily assessment until discharge to evaluate postoperative recovery and complications. All enrolled patients underwent appendectomy during the index admission according to institutional treatment protocols and surgeon discretion. No patients were managed conservatively with interval appendectomy.

Statistical analysis

Data were entered into Microsoft Excel 2016 (Microsoft Corp., Redmond, WA, USA) and analyzed using IBM SPSS Statistics for Windows, Version 20 (Released 2011; IBM Corp., Armonk, New York, United States). Continuous variables were expressed as mean ± standard deviation, while categorical variables were presented as frequencies and percentages. Chi-square test and independent sample t-test were used for comparisons. ROC curve analysis was performed to assess the diagnostic accuracy of serum sodium, CRP, and TLC. Correlation analysis was used to evaluate the relationship between biomarkers and hospital stay. A p-value <0.05 was considered statistically significant.

## Results

The present study included 72 patients diagnosed with acute appendicitis. The mean age of the study population was 41.1±15.26 years, with an age range of 18-78 years, indicating a wide age distribution. The largest proportion of patients belonged to the 31-40 years age group (n=23; 31.9%), followed by patients aged >50 years (n=20; 27.8%), 41-50 years (n=11; 15.3%), and 21-30 years (n=10; 13.9%). Patients aged ≤20 years (n=8; 11.1%) represented the smallest subgroup. These findings indicate that acute appendicitis was more commonly observed among middle-aged individuals in the present study (Table 1).

**Table 1:** Baseline demographic and clinical characteristics of study participants (N=72) HPE: Histopathological Examination.

Parameter	Domain	n (%) / Mean ± SD
Age distribution	Age (years), mean±SD	41.1±15.26
	Age range (years)	18–78
	≤20 years	8 (11.1)
	21–30 years	10 (13.9)
	31–40 years	23 (31.9)
	41–50 years	11 (15.3)
	>50 years	20 (27.8)
Gender	Male	49 (68.1)
	Female	23 (31.9)
Clinical diagnosis	Acute appendicitis	57 (79.2)
	Perforated appendix	11 (15.3)
	Recurrent appendicitis	4 (5.6)
HPE diagnosis	Acute appendicitis	60 (83.3)
	Gangrenous appendix	1 (1.4)
	Gangrenous appendix with perforation	11 (15.3)

Gender distribution demonstrated a predominance of male patients, with 49 (68.1%) male subjects and 23 (31.9%) female subjects, corresponding to an approximate male-to-female ratio of 2.1:1 (Table 1).

Regarding clinical diagnosis, the majority of patients had acute appendicitis (n=57; 79.2%), while a perforated appendix was identified in 11 patients (15.3%), suggesting complicated disease presentation. Recurrent appendicitis was observed in 4 patients (5.6%). Histopathological examination findings were largely consistent with clinical diagnoses and confirmed acute appendicitis in 60 patients (83.3%). Severe pathological changes included gangrenous appendix with perforation in 11 patients (15.3%) and an isolated gangrenous appendix in one patient (1.4%). These findings suggest that uncomplicated acute appendicitis constituted the predominant clinical presentation; however, a considerable proportion of patients presented with advanced disease pathology (Table 1).

Ultrasonography findings demonstrated acute appendicitis in 59 patients (81.9%), making it the most common radiological diagnosis. Subacute appendicitis was observed in six patients (8.3%), while perforated appendix was identified in four patients (5.6%). Other findings included mesenteric lymphadenopathy in one (1.4%), pelvic inflammatory disease (PID) in one (1.4%), and normal ultrasonographic findings in one (1.4%) patient each. These findings indicated a high diagnostic yield of ultrasonography for identifying appendiceal pathology in the study population (Table 2).

**Table 2:** Imaging findings, intraoperative findings, and surgical procedures USG: Ultrasonography; PID: Pelvic Inflammatory Disease.

Parameter	Domain	n (%)
USG findings	Acute appendicitis	59 (81.9)
	Subacute appendicitis	6 (8.3)
	Perforated appendix	4 (5.6)
	Mesenteric lymphadenopathy	1 (1.4)
	PID	1 (1.4)
	Normal study	1 (1.4)
Intraoperative findings	Inflamed appendix	47 (65.3)
	Perforated appendix	11 (15.3)
	Dilated appendix with thick wall	8 (11.1)
	Edematous appendix	5 (6.9)
	Terminal part sloughed	1 (1.4)
Surgical procedures	Open appendectomy	56 (77.8)
	Laparoscopic appendectomy	13 (18.1)
	Diagnostic laparoscopy + appendectomy	1 (1.4)
	Exploratory laparotomy + appendectomy + ileostomy	1 (1.4)
	Laparoscopic converted to open	1 (1.4)

Intraoperative findings revealed that an inflamed appendix was the most common observation, noted in 47 patients (65.3%). Perforated appendix was identified in 11 patients (15.3%), while dilated appendix with thickened wall was observed in eight patients (11.1%). Additional findings included edematous appendix in five patients (6.9%) and terminal appendiceal sloughing in one patient (1.4%), suggesting varying degrees of disease severity and progression (Table 2).

Regarding surgical management, open appendectomy was the predominant procedure performed in 56 patients (77.8%), whereas laparoscopic appendectomy was undertaken in 13 patients (18.1%). Complex or modified surgical procedures were required in a small proportion of cases, including diagnostic laparoscopy followed by appendectomy in one (1.4%), exploratory laparotomy with appendectomy and ileostomy in one (1.4%), and conversion from laparoscopic to open appendectomy in one (1.4%) patient each. These findings suggest that open appendectomy remained the preferred surgical approach in the present study (Table 2).

The comparison of demographic characteristics between non-perforated and perforated appendicitis groups demonstrated a significant association between age and disease severity. Patients with perforated appendicitis had a significantly higher mean age (49.55±18.30 years) compared with those with non-perforated appendicitis (39.59±14.31 years) (t = -2.03; 95% CI: −19.72 to −0.20; p=0.046), suggesting that increasing age may be associated with a greater risk of perforation (Table 3).

**Table 3:** Comparison of demographic variables and hyponatremia between non-perforated and perforated appendicitis

Variable	Non-perforated (N=61)	Perforated (N=11)	Test statistic	95% CI	P-value
Age (years), mean±SD	39.59±14.31	49.55±18.30	t = -2.03	−19.72 to −0.20	0.046
Male, n (%)	43 (70.5)	6 (54.5)	OR = 0.50	0.14–1.86	0.296
Female, n (%)	18 (29.5)	5 (45.5)	Reference	—	—
Hyponatremia (<135 mEq/L), n (%)	5 (8.2)	7 (63.6)	OR = 19.60	4.24–90.67	<0.0001

Gender distribution did not demonstrate a statistically significant association with appendiceal perforation. Male subjects constituted 43 patients (70.5%) in the non-perforated group and six patients (54.5%) in the perforated group (OR=0.50; 95% CI: 0.14-1.86; p=0.296), indicating that sex was not a significant predictor of disease severity in the present study (Table 3).

Hyponatremia (<135 mEq/L) showed a highly significant association with appendiceal perforation. Hyponatremia was present in only five patients (8.2%) in the non-perforated group compared with seven patients (63.6%) in the perforated group. Patients with hyponatremia had approximately 19.6-fold higher odds of perforation (OR=19.60; 95% CI: 4.24-90.67; p<0.0001), highlighting serum sodium as a potentially valuable marker for predicting complicated appendicitis (Table 3).

Comparison of electrolyte parameters between non-perforated and perforated appendicitis demonstrated a significant reduction in serum sodium levels among patients with perforated appendicitis (133.63±3.80 mEq/L) compared with non-perforated cases (137.92±2.88 mEq/L) (t=4.29; 95% CI: 2.33 to 6.25; p<0.001). In contrast, serum potassium (4.00±0.25 vs. 4.13±0.35 mEq/L; t=1.17; 95% CI: −0.09 to 0.35; p=0.245), chloride (101.73±3.20 vs. 101.71±3.20 mEq/L; t=−0.02; 95% CI: −2.15 to 2.11; p=0.989), and bicarbonate levels (24.64±2.25 vs. 25.48±3.00 mEq/L; t=0.89; 95% CI: −1.04 to 2.72; p=0.381) did not differ significantly between the groups (Table 4).

**Table 4:** Comparison of laboratory parameters between non-perforated and perforated appendicitis ALP: Alkaline Phosphatase; SGPT: Serum Glutamic Pyruvic Transaminase; SGOT: Serum Glutamic Oxaloacetic Transaminase; CRP: C-reactive protein; TLC: Total Leukocyte Count.

Parameters	Domain	Non-perforated (N=61); Mean±SD	Perforated (N=11); Mean±SD	t-value	95% CI of Mean Difference	P-value
Electrolytes	Serum sodium (mEq/L)	137.92±2.88	133.63±3.80	4.29	2.33 to 6.25	<0.001
	Serum potassium (mEq/L)	4.13±0.35	4.00±0.25	1.17	−0.09 to 0.35	0.245
	Chloride (mEq/L)	101.71±3.20	101.73±3.20	−0.02	−2.15 to 2.11	0.989
	HCO3 (mEq/L)	25.48±3.00	24.64±2.25	0.89	−1.04 to 2.72	0.381
Renal function	Blood urea (mg/dL)	25.09±9.53	28.82±6.71	−1.25	−9.78 to 2.32	0.220
	Serum creatinine (mg/dL)	0.75±0.22	0.74±0.22	0.25	−0.07 to 0.09	0.807
Liver function	Total bilirubin (mg/dL)	1.37±0.83	1.70±0.50	−1.28	−0.84 to 0.18	0.205
	Direct bilirubin (mg/dL)	0.32±0.15	0.47±0.19	−3.04	−0.25 to −0.05	0.003
	Albumin (g/dL)	4.43±0.66	4.43±0.87	0.00	−0.51 to 0.51	0.998
	Globulin (g/dL)	3.21±0.70	3.25±0.32	−0.19	−0.47 to 0.39	0.887
	Total protein (g/dL)	7.20±0.58	6.59±0.87	2.99	0.21 to 1.01	0.004
	ALP (U/L)	106.33±84.97	107.00±21.60	−0.03	−42.87 to 41.53	0.979
	SGOT (U/L)	27.67±13.22	30.55±9.03	−0.81	−9.95 to 4.19	0.492
	SGPT (U/L)	28.15±16.25	29.27±9.52	−0.22	−11.16 to 8.92	0.825
Inflammatory and metabolic markers	TLC (×10³/µL)	10.89±3.34	12.69±1.73	−1.73	−3.89 to 0.29	0.086
	CRP (mg/dL)	5.50±4.29	14.41±9.16	−5.23	−12.58 to −5.24	<0.001
	Random blood sugar (mg/dL)	97.08±15.74	109.27±29.51	−2.03	−24.22 to −0.16	0.046

Renal function parameters including blood urea and serum creatinine showed no statistically significant differences between groups. Blood urea levels were 28.82±6.71 mg/dL in perforated cases compared with 25.09±9.53 mg/dL in non-perforated cases (t=−1.25; 95% CI: −9.78 to 2.32; p=0.220), while serum creatinine levels remained comparable (0.74±0.22 vs. 0.75±0.22 mg/dL; t=0.25; 95% CI: −0.07 to 0.09; p=0.807) (Table 4).

Among liver function parameters, direct bilirubin levels were significantly higher in perforated appendicitis (0.47±0.19 mg/dL) than non-perforated appendicitis (0.32±0.15 mg/dL) (t=−3.04; 95% CI: −0.25 to −0.05; p=0.003). Total protein levels were significantly lower in perforated cases (6.59±0.87 g/dL) compared with non-perforated cases (7.20±0.58 g/dL) (t=2.99; 95% CI: 0.21 to 1.01; p=0.004). However, total bilirubin, albumin, globulin, alkaline phosphatase (ALP), serum glutamic pyruvic transaminase (SGPT) and serum glutamic oxaloacetic transaminase (SGOT) did not demonstrate statistically significant differences between the groups (Table 4).

Analysis of inflammatory and metabolic markers demonstrated markedly elevated CRP levels among patients with perforated appendicitis (14.41±9.16 mg/dL) compared with non-perforated cases (5.50±4.29 mg/dL) (t=−5.23; 95% CI: −12.58 to −5.24; p<0.001). Random blood sugar levels were also significantly higher in perforated cases (109.27±29.51 mg/dL) than in non-perforated cases (97.08±15.74 mg/dL) (t=−2.03; 95% CI: −24.22 to −0.16; p=0.046). Although TLC was higher in the perforated group (12.69±1.73 ×10³/µL) compared with the non-perforated group (10.89±3.34 ×10³/µL), the difference did not reach statistical significance (t=−1.73; 95% CI: −3.89 to 0.29; p=0.086) (Table 4).

**Figure 1:**
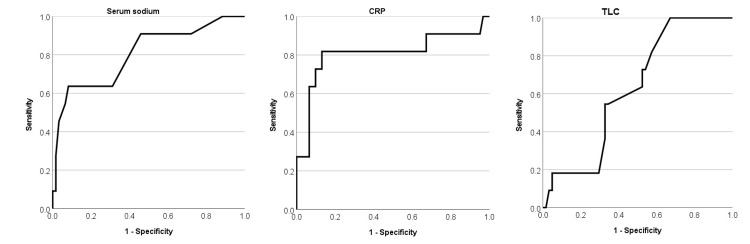
ROC curve analysis for serum sodium, CRP, and TLC CRP: C-reactive protein; TLC: Total Leukocyte Count; ROC: Receiver Operating Characteristic.

ROC analysis was performed to evaluate the diagnostic performance of various biomarkers for predicting perforated appendicitis. Among the biomarkers studied, CRP demonstrated the highest discriminatory ability with an area under the curve or AUC of 0.806 (95% CI: 0.622-0.991; p=0.001), indicating good predictive performance. At an optimal cut-off value of 8.8 mg/dL, CRP showed a sensitivity of 81.8% and specificity of 86.9%, suggesting balanced diagnostic accuracy for identifying perforated appendicitis (Table 5 and Figure 1).

**Table 5:** ROC analysis of biomarkers for prediction of perforated appendicitis CRP: C-reactive protein; TLC: Total Leukocyte Count; ROC: Receiver Operating Characteristic.

Biomarker	AUC	95% CI	Sensitivity (%)	Specificity (%)	Cut-off	P-value
CRP	0.806	0.622–0.991	81.8	86.9	8.8	0.001
Serum sodium	0.803	0.648–0.959	63.6	91.8	135	0.001
TLC	0.626	0.478–0.774	54.5	67.2	12.3	0.186

Serum sodium demonstrated good predictive ability with an AUC of 0.803 (95% CI: 0.648-0.959; p=0.001). At a cut-off value of 135 mEq/L, serum sodium showed sensitivity of 63.6% and specificity of 91.8% for predicting perforated appendicitis. These findings suggest that serum sodium may serve as a useful biomarker for identifying complicated appendicitis (Table 5 and Figure 1).

In contrast, TLC showed comparatively poor discriminatory performance with an AUC of 0.626 (95% CI: 0.478-0.774; p=0.186), which was statistically non-significant. At a cut-off value of 12.3×10³/µL, TLC demonstrated only 54.5% sensitivity and 67.2% specificity, indicating limited utility in predicting perforation compared with CRP and serum sodium (Table 5 and Figure 1).

Comparison of clinical outcomes between non-perforated and perforated appendicitis demonstrated a significant difference in hospital stay duration. Patients with perforated appendicitis had a significantly longer mean hospital stay (5.0±2.14 days) compared with patients with non-perforated appendicitis (3.44±1.09 days) (t=−3.44; 95% CI: −2.46 to −0.66; p<0.001), indicating increased disease severity and greater healthcare utilization in complicated cases (Table 6).

**Table 6:** Hospital stay and complications

Parameter	Non-perforated (N=61)	Perforated (N=11)	Test statistic	95% CI	P-value
Length of hospital stay (days), Mean±SD	3.44±1.09	5.0±2.14	t = −3.44	−2.46 to −0.66	<0.001
Any postoperative complications, n (%)	2 (3.3)	5 (45.5)	OR = 24.58	3.96–152.57	<0.0001

Postoperative complications were significantly more frequent among patients with perforated appendicitis. Complications occurred in five patients (45.5%) in the perforated group compared with only two patients (3.3%) in the non-perforated group. Patients with perforated appendicitis had approximately 24.58-fold higher odds of developing postoperative complications (OR=24.58; 95% CI: 3.96-152.57; p<0.0001), highlighting the substantial increase in adverse postoperative outcomes associated with appendiceal perforation (Table 6).

Correlation analysis was performed to evaluate the relationship between biomarkers and duration of hospital stay. Serum sodium demonstrated a significant negative correlation with hospital stay (r = −0.261; p=0.027), indicating that lower serum sodium levels were associated with prolonged hospitalization. This finding suggests that hyponatremia may be associated with increased disease severity and delayed recovery (Table 7).

**Table 7:** Correlation of biomarker with hospital stay CRP: C-reactive protein; TLC: Total Leukocyte Count.

Biomarker	Correlation coefficient (r)	P-value
Serum sodium	−0.261	0.027
CRP	0.249	0.035
TLC	0.053	0.659

In contrast, CRP showed a significant positive correlation with hospital stay (r = 0.249; p=0.035), suggesting that higher inflammatory burden was associated with longer duration of hospitalization (Table 7).

However, TLC demonstrated only a weak and statistically non-significant positive correlation with hospital stay (r = 0.053; p=0.659), indicating limited predictive value for duration of hospitalization in the present study (Table 7).

ROC analysis was performed to evaluate the ability of serum sodium to predict postoperative complications. Serum sodium demonstrated acceptable discriminatory performance with an AUC of 0.733 (95% CI: 0.554-0.912; p=0.044), indicating statistically significant predictive ability (Table 8).

**Table 8:** ROC analysis of serum sodium for prediction of postoperative complications ROC: Receiver Operating Characteristic.

Parameter	AUC	95% CI	Sensitivity (%)	Specificity (%)	Cut-off	P-value
Serum sodium	0.733	0.554–0.912	85.7	50.8	135	0.044

At the optimal cut-off value of 135 mEq/L, serum sodium showed a high sensitivity of 85.7% and a specificity of 50.8% for predicting postoperative complications. These findings suggest that lower serum sodium levels may serve as a useful marker for identifying patients at increased risk of postoperative adverse outcomes, particularly due to its high sensitivity in detecting at-risk individuals (Table 8).

## Discussion

This prospective study evaluated serum sodium as a predictive biomarker for appendiceal perforation in 72 adult patients with acute appendicitis and compared its diagnostic performance with CRP and TLC. The study also assessed the relationship between these biomarkers and clinical outcomes, including hospital stay and postoperative complications.

The study population had a mean age of 41.1±15.26 years, with most patients in the 31 to 40-year age group. Male subjects constituted 68.1% of the cohort, yielding a male-to-female ratio of 2.1:1. This male predominance is consistent with previous reports by Turhan et al. and Maqbool et al., who documented male patient proportions of 56.7% and 60%, respectively [11,12]. However, Symeonidis et al. observed a predominance of female patients, suggesting demographic variability among populations [13].

A significant association was observed between increasing age and appendiceal perforation. Patients with perforated appendicitis had a significantly higher mean age than non-perforated cases (49.55±18.30 vs. 39.59±14.31 years; p=0.046). Similar findings were reported by Senol et al. [14], who observed a higher mean age among patients with complicated appendicitis, while Wu et al. [15] also demonstrated increased perforation rates in older patients. These findings suggest that advancing age may contribute to delayed diagnosis, atypical presentations, or greater disease severity.

One of the principal findings of the present study was the strong association between hyponatremia and appendiceal perforation. Patients with perforated appendicitis demonstrated significantly lower serum sodium levels than non-perforated patients (133.63±3.8 vs. 137.92±2.88 mEq/L; p<0.001). Hyponatremia (<135 mEq/L) was associated with nearly 20-fold higher odds of perforation. Similar findings have been consistently reported in previous studies. Senol et al. [14] reported an AUC of 0.727, while Messias et al. [9] observed an AUC of 0.696. Shuaib et al. [16] and Maqbool et al. [12] similarly supported serum sodium as a marker of complicated appendicitis.

ROC analysis in the present study demonstrated good discriminatory ability of serum sodium (AUC=0.803; 95% CI: 0.648-0.959; p=0.001). At a cut-off of 135 mEq/L, serum sodium showed sensitivity of 63.6% and high specificity of 91.8% for predicting perforated appendicitis. These findings suggest that serum sodium may be particularly useful for identifying patients at higher risk of complicated appendicitis. However, its modest sensitivity indicates that approximately one-third of perforated cases may not be identified using this cutoff alone. Therefore, serum sodium should be considered an adjunctive biomarker and interpreted alongside clinical findings and other inflammatory markers such as CRP.

The pathophysiological mechanism of hyponatremia in complicated appendicitis is thought to involve systemic inflammation. Cytokines, particularly interleukin-6, stimulate non-osmotic secretion of antidiuretic hormone, leading to water retention and dilutional hyponatremia. Lindestam et al. demonstrated elevated plasma vasopressin levels in perforated appendicitis, supporting this mechanism [17]. Thus, serum sodium may reflect systemic inflammatory response rather than isolated local disease.

Among evaluated biomarkers, CRP demonstrated the best overall diagnostic performance (AUC=0.806), with sensitivity of 81.8% and specificity of 86.9%. Similar observations have been reported by Wu et al. [15] and Kim et al. [6], who found strong correlations between CRP and disease severity. Conversely, TLC showed poor discriminatory performance (AUC=0.626; p=0.186), consistent with findings by Kim et al. [6] and Turhan et al. [11], suggesting that TLC alone may have limited utility for predicting disease severity. These findings indicate that serum sodium and CRP may have complementary roles, with serum sodium demonstrating higher specificity and CRP demonstrating higher sensitivity.

Serum sodium also demonstrated prognostic utility beyond diagnosis. Patients with perforated appendicitis had significantly longer hospital stays (5.0±2.14 vs. 3.44±1.09 days; p<0.001). Serum sodium showed a significant negative correlation with hospital stay (r=−0.261; p=0.027), while CRP demonstrated a positive correlation (r=0.249; p=0.035). These findings suggest that lower sodium levels and higher inflammatory burden are associated with prolonged recovery. Similar findings were reported by Wu et al. [15].

Postoperative complications occurred significantly more frequently in perforated appendicitis (45.5% vs. 3.3%; p<0.0001). Serum sodium also predicted postoperative complications with acceptable discriminatory ability (AUC=0.733; p=0.044), suggesting potential value in perioperative risk stratification. However, because only seven postoperative complications occurred in the study cohort, these findings should be interpreted cautiously and require validation in larger studies.

The strengths of this study include its prospective design, histopathological confirmation of diagnosis, standardized evaluation of all enrolled patients, and simultaneous comparison of serum sodium with established inflammatory biomarkers such as CRP and TLC within the same cohort. This study has several limitations. First, it was conducted at a single tertiary care center with a relatively small sample size, which may limit the generalizability of the findings to other populations and healthcare settings. The sample size calculation was based on sensitivity estimates derived from a pediatric study due to the limited availability of prospective adult data at the time of study planning. Second, the number of patients with perforated appendicitis and postoperative complications was limited, which may have reduced the precision of subgroup analyses and diagnostic performance estimates. In particular, the postoperative complication analysis was based on a small number of events, and therefore the corresponding ROC findings should be interpreted with caution. Third, patients with perforated appendicitis were significantly older than those without perforation, raising the possibility of residual confounding by age, as age may independently influence both disease severity and serum sodium levels. Fourth, the single-center design may have introduced selection bias despite the prospective recruitment of consecutive eligible patients. Finally, serial measurements of serum sodium and inflammatory markers were not performed; therefore, the temporal relationship between biomarker changes and disease progression could not be evaluated. Larger multicenter studies with more diverse populations and greater numbers of complicated cases are required to validate these findings.

## Conclusions

The present study suggests that serum sodium is a simple, inexpensive, and readily available adjunctive biomarker associated with appendiceal perforation in patients with acute appendicitis. Hyponatremia (<135 mEq/L) was strongly associated with complicated disease and demonstrated good diagnostic utility for identifying perforated appendicitis. Lower serum sodium levels were also associated with prolonged hospital stay and increased postoperative complications, highlighting its prognostic relevance. Although CRP demonstrated superior overall diagnostic accuracy, serum sodium showed high specificity and may serve as a useful adjunctive biomarker for identifying complicated appendicitis. Serum sodium should be considered as part of a multimodal assessment rather than a standalone diagnostic tool. Given the relatively small sample size and single-center design, larger multicenter studies are required to validate these findings before routine clinical implementation can be recommended.

## References

[1] Gomes CA, Sartelli M, Di Saverio S (2015). Acute appendicitis: proposal of a new comprehensive grading system based on clinical, imaging and laparoscopic findings. World J Emerg Surg.

[2] Patel K, Bhasin G, Lasday M, Plamoottil A, Ganti L (2025). Acute appendicitis masquerading as food poisoning. Radiol Case Rep.

[3] Ben Ismail I, Amari R, Zenaidi H, Sghaier M, Rebii S, Zoghlami A (2026). Preoperative prediction of complicated acute appendicitis using the appendicitis inflammatory response (AIR) score. Curr Probl Surg.

[4] Di Saverio S, Podda M, De Simone B (2020). Diagnosis and treatment of acute appendicitis: 2020 update of the WSES Jerusalem guidelines. World J Emerg Surg.

[5] Bom WJ, Scheijmans JC, Salminen P, Boermeester MA (2021). Diagnosis of uncomplicated and complicated appendicitis in adults. Scand J Surg.

[6] Kim DY, Nassiri N, de Virgilio C, Ferebee MP, Kaji AH, Hamilton CE, Saltzman DJ (2015). Association between hyponatremia and complicated appendicitis. JAMA Surg.

[7] Singla S, Chanderbhan Chanderbhan, Pranesh M, Kesharwani S, Garg R, Pandey SB (2025). Role of hyponatremia as a predictive biomarker for complicated acute appendicitis: a comparative clinical study. Eur J Cardiovasc Med.

[8] Farooqui W, Pommergaard HC, Burcharth J, Eriksen JR (2015). The diagnostic value of a panel of serological markers in acute appendicitis. Scand J Surg.

[9] Messias B, Cubas I, Oliveira C (2023). Usefulness of serum sodium levels as a novel marker for predicting acute appendicitis severity: a retrospective cohort study. BMC Surg.

[10] Elgendy A, Khirallah MG, Elsawaf M, Hassan HS, Ghazaly M (2023). Acute appendicitis in children: is preoperative hyponatremia a predictive factor of perforation/gangrene? A prospective study. Pediatr Surg Int.

[11] Turhan VB, Ünsal A, Öztürk B, Öztürk D, Buluş H (2022). Predictive value of serum sodium level in determining perforated appendicitis. Ulus Travma Acil Cerrahi Derg.

[12] Maqbool M, Khaliq F, Mehboob A, Anis S, Hussain M, Amjad M (2022). Diagnostic value of serum urea, creatinine, sodium and potassium for complicated appendicitis - a one year retrospective study. J Ayub Med Coll Abbottabad.

[13] Symeonidis NG, Pavlidis ET, Psarras KK (2022). Preoperative hyponatremia indicates complicated acute appendicitis. Surg Res Pract.

[14] Senol S, Kusak M, Özdemir DB, Sendil AM (2024). Diagnostic value of serum sodium level and neutrophil-to-lymphocyte ratio in predicting severity of acute appendicitis: a retrospective cross-sectional two-center study. Medicina (Kaunas).

[15] Wu HC, Yan MT, Lu KC, Chu P, Lin SH, Yu JC, Wu CC (2013). Clinical manifestations of acute appendicitis in hemodialysis patients. Surg Today.

[16] Shuaib A, Alhamdan N, Arian H, Sallam MA, Shuaib A (2022). Hyperbilirubinemia and hyponatremia as predictors of complicated appendicitis. Med Sci (Basel).

[17] Lindestam U, Norberg Å, Svensson JF (2026). Plasma sodium as a predictor of perforation in acute appendicitis: a prospective multi-centre study. J Pediatr Surg.

